# Combination of Intravitreal Bevacizumab and Peripheral Photocoagulation: An Alternative Treatment in Eales Disease

**Published:** 2013

**Authors:** Juarez CP, Gramajo AL, Luna JD

**Affiliations:** Department of Ophthalmology, Centro de Ojos Romagosa-Fundación VER, Córdoba, Argentina

**Keywords:** Bevacizumab, Eales disease, Retinal neovascularization, Retinal photocoagulation

## Abstract

To report the efficacy of combination therapy (bevacizumab and photocoagulation) in a case of Eales Disease this study has been performed. Bevacizumab (Avastin, 1.25 mg/0.05 ml) was injected intravitreously for the treatment of iris and retinal neovascularization in a 56-year old Hispanic female with photocoagulation treatment to control the recurrence of vitreous haemorrhage.

Our results revealed that stabilization of the disease and improvement in visual acuity were achieved without any signs of recurrence. Intravitreal bevacizumab in combination with photocoagulation treatment of ischemic retinal areas may be a good alternative for patients with recurrent vitreous haemorrhage due to Eales disease.

## INTRODUCTION

Eales disease (ED) is a vasoproliferative retinal disease characterized by progressive peripheral retinal capillary closure resulting in increasing retinal non-perfusion, ischemia, and neovascularization. The pathogenesis of ED has not completely understood. However, it involves extensive retinal ischemia, resulting in the release of angiogenic factors such as the vascular endothelial growth factor (VEGF) [1,2].

Bevacizumab (Avastin®), a full-length humanized monoclonal antibody that binds to all VEGF-A isoforms is, at present, one of the most effective treatments for neovascular conditions such as age-related macular degeneration and proliferative diabetic retinopathy. We report the a 56-year old female with rubeosis iridis and retinal neovascularization due to ED who was treated with a combination of retinal photocoagulation and intraocular bevacizumab (Avastin®).

## CASE REPORT

A 56-year old Hispanic woman experiencing blurred vision in her right eye consulted our retina department in August 2006. She had previously suffered from vitreous hemorrhage which had resolved spontaneously in both eyes for 3 years duration. At the time of her first visit, she was treated with beta-blockers and prostaglandin analog eye drops for bilateral chronic open angle glaucoma diagnosed 8 years ago. Her corrected visual acuity (BCVA) was 20/60 in her right eye (OD) and 20/30 in the left one (OS). Slit lamp biomiscroscopy, revealed the presence of tiny, neovascular, dilated capillary tufts at the pupillary margin (rubeosis iridis) in her OD. The intraocular pressure (IOP) has been measured within normal limits in both eyes (16 mmHg). Dilated fundus examination revealed a peripheral vascular sheating at the temporal vascular zone in both eyes. Fine solid white lines, representing obliterated larger vessels as well as vitreous hemorrhage were detected in the right eye.

**Figure 1:**
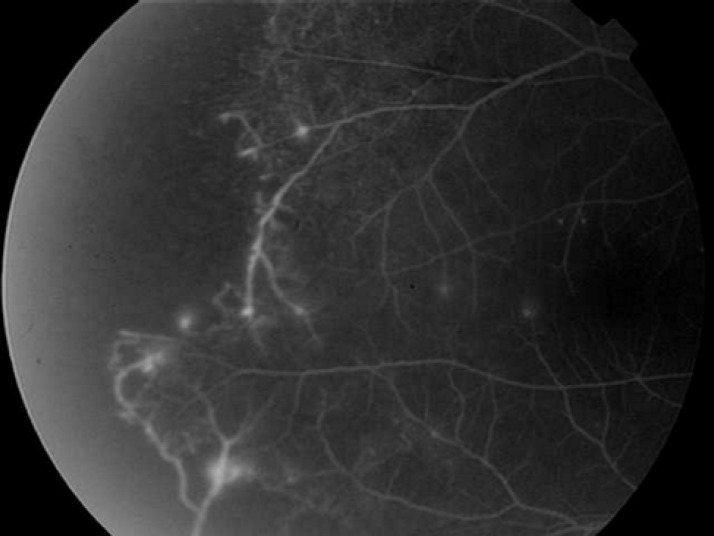
Fluorescein angiography shows leakage from the suspected new vessels with peripheral capillary non-perfusion

Retinal neovascularization visualized bilaterally and retinal scars from previous laser photocoagulation treatment were present in areas of the temporal vascular retina in both eyes. Leakage from suspected new vessels together with peripheral capillary nonperfusion area was noted in fluorescein angiography (Figure 1). A diagnostic laboratory evaluation was performed including: routine tests (complete blood count, erythrocyte sedimentation rate, blood sugar, protein electrophoresis, blood chemistry, chest X-ray, urinalysis, C- reactive protein), infectious disease laboratory (VDRL, FTA-ABS, PPD and Brucella titer) and systemic autoimmune disorder tests (antinuclear antibody, anti-double stranded DNA antibodies, ANCA, C3 and C4). All of the values were within normal limits. 

A mantoux test showed lightly positive result. Since in Argentina almost all citizens have been immunized for TB with a BCG vaccination, we considered this measurement as a negative value. The patient was diagnosed as Eales disease due to the absence of a cause for the retinal vascular disease. 

As the patient preferred not to undergo a new proposed laser treatment in the OD, an intravitreous bevacizumab (1.25 mg/0.05 ml) (Avastin® Roche Argentina) was applied. The treatment was carried out and subsequently almost seven days following the procedure an improvement in the iris and retinal neovascularization with the improvement in vision to 20/25 has been noticed. Subsequently, in March 2007 she presented with a vitreous hemorrhage in the contralateral eye. The visual acuity dropped to 20/70 and she has received an intravitreal Avastin® in the OS. A subsequent improvement of her visual acuity was initially detected but 2 months later recurrent hemovitreous had returned to the same eye. A second application of bevacizumab (Avastin®) was administered. This time, seven days after bevacizumab application, laser photocoagulation treatment was performed over the ischemic areas of the left eye. After four years follow-up her BCVA was detected to 20/30 OD and 20/70 OS with regressed rubeosis iridis, IOP readings of 18 (OD) and 17 mmHg (OS) and mild nuclear sclerosis. A fundus examination revealed no retinal neovascularization or vitreous hemorrhage. Furthermore, due to financial difficulties either OCT or new Angiography were not performed. The follow-up was performed every 6 months and she did not present any sign of other problems at the time of visit.

## DISCUSSION

ED is an idiopathic obliterative vasculopathy. Clinical findings are characterized by avascular areas in the retina periphery followed posteriorly by microaneurysms, dilatation of capillary channels, tortuosity of neighboring vessels, and spontaneous chorioretinal scars [3]. It is a diagnosis of exclusion, as many other retinal disorders can mimic ED, especially conditions of retinal inflammation or neovascularization. Visual compromise is seen in patients with recurrent vitreous hemorrhage, but resolves to better than 20/200 in greater than 70% of patients. If retinal nonperfusion extends into the macula, the visual acuity usually is worse than 20/400. Often, patients complain of uniocular symptoms, but ophthalmic examination reveals changes of ED in the other eye like our patient. Bilateral involvement is evident in 80-90% of patients. Neovascularization of the disc (NVD) or neovascularization elsewhere (NVE) was observed in up to 80% of patients with ED [4]. 

The NVE usually is located peripherally, at the junction of perfused and nonperfused retina. The neovascularization often is the source of vitreous hemorrhage in these eyes, compromising vision. Rubeosis iridis or neovascularization of the iris can develop and may lead to neovascular glaucoma. The pathogenesis of ED involves extensive retinal ischemia resulting in the angiogenic factors, such as IL-6, IL-8, MCP-1, and VEGF, which were previously found in the vitreous of patients with Eales disease by Murugeswari et al [5]. 

Neovascularization from the disc (NVD) or elsewhere (NVE) in the retina is observed in up to 80% of patients with ED, with retinal neovascularization often being the source of vitreous hemorrhage in these eyes. Current treatment options for ED include intraocular steroids [6], retinal photocoagulation to the non-perfused retina, and early vitrectomy for recurrent vitreous hemorrhage [7]. However, some recent articles have suggested intraocular bevacizumab as a new form of treatment in neovascular ED [8,9,10] or as a possible adjunctive treatment to vitreoretinal surgery for the management of ED with tractional retinal detachment [11].

However, this treatment has a legacy of serious complications. Recently, Kumar A et al reported four out of fourteen patients who had received pretreatment with intravitreal bevacizumab (0.25 mg) and subsequently underwent a pars plana vitrectomy for non-resolving vitreous hemorrhage and/or tractional retinal detachment developed secondary rhegmatogenous retinal detachment, within a week of receiving intravitreal bevacizumab [12]. Similar results were found recently by Patwardhan SD, et al [13]. It seems this kind of complication could happen also to patients with Eales along with vitreous hemorrhage or mild bleeding. But these kinds of complications presumably could be avoided if laser photocoagulation was made prior to intraocular bevacizumab treatment in the entire peripheral ischemic retina. In our patient, intravitreal anti-VEGF therapy alone was not sufficient to control the disease and combination therapy of laser treatment and intraocular bevacizumab was effective in improving and stabilizing her vision. 

In conclusion, our case suggests that combined laser therapy and intraocular anti-VEGF therapy may be necessary for the control of neovascularization, leading to an improvement of visual acuity in patients with complicated ED. A close follow-up for the early detection of recurrent neovascularization and subsequent prompt treatment may improve the visual outcomes in such patients. Furthermore, more trials should be conducted to confirm the safety and efficacy of this treatment module.

## DISCLOSURE

Conflicts of Interest: None declared.
